# Machine Learning for Early Warning of Septic Shock in Children With Hematological Malignancies Accompanied by Fever or Neutropenia: A Single Center Retrospective Study

**DOI:** 10.3389/fonc.2021.678743

**Published:** 2021-06-15

**Authors:** Long Xiang, Hansong Wang, Shujun Fan, Wenlan Zhang, Hua Lu, Bin Dong, Shijian Liu, Yiwei Chen, Ying Wang, Liebin Zhao, Lijun Fu

**Affiliations:** ^1^ Department of Pediatrics Intensive Care Unit, Shanghai Children’s Medical Center, School of Medicine, Shanghai Jiao Tong University, Shanghai, China; ^2^ Pediatric Artificial Intelligence Clinical Application and Research Center, Shanghai, China; ^3^ Shanghai Engineering Research Center of Intelligence, Shanghai, China; ^4^ Child Health Advocacy Institute, China Hospital Development Institute of Shanghai Jiao Tong University, Shanghai, China; ^5^ Medical Affairs, Shanghai Synyi Medical Technology CO., Ltd, Shanghai, China; ^6^ School of Public Health, Shanghai Jiao Tong University, Shanghai, China; ^7^ Department of Cardiology, Shanghai Children’s Medical Center, School of Medicine, Shanghai Jiao Tong University, Shanghai, China

**Keywords:** machine learning, predictive modeling, hematological or oncological disease, fever, neutropenia, septic shock

## Abstract

**Objectives:**

The purpose of this article was to establish and validate clinically applicable septic shock early warning model (SSEW model) that can identify septic shock in hospitalized children with onco-hematological malignancies accompanied with fever or neutropenia.

**Methods:**

Data from EMRs were collected from hospitalized pediatric patients with hematological and oncological disease at Shanghai Children’s Medical Center. Medical records of patients (>30 days and <19 years old) with fever (≥38°C) or absolute neutrophil count (ANC) below 1.0 × 10^9^/L hospitalized with hematological or oncological disease between January 1, 2017 and August 1, 2019 were considered. Patients in whom septic shock was diagnosed during the observation period formed the septic shock group, whereas non-septic-shock group was the control group. In the septic shock group, the time points at 4, 8, 12, and 24 hours prior to septic shock were taken as observation points, and corresponding observation points were obtained in the control group after matching. We employed machine learning artificial intelligence (AI) to filter features and used XGBoost algorithm to build SSEW model. Area under the ROC curve (AU-ROC) was used to compare the effectiveness among the SSEW Model, logistic regression model, and pediatric sequential organ failure score (pSOFA) for early warning of septic shock.

**Main Results:**

A total of 64 observation periods in the septic shock group and 2191 in the control group were included. AU-ROC of the SSEW model had higher predictive value for septic shock compared with the pSOFA score (0.93 vs. 0.76, Z = −2.73, P = 0.006). Further analysis showed that the AU-ROC of the SSEW model was superior to the pSOFA score at the observation points 4, 8, 12, and 24 h before septic shock. At the 24 h observation point, the SSEW model incorporated 14 module root features and 23 derived features.

**Conclusion:**

The SSEW model for hematological or oncological pediatric patients could help clinicians to predict the risk of septic shock in patients with fever or neutropenia 24 h in advance. Further prospective studies on clinical application scenarios are needed to determine the clinical utility of this AI model.

## Introduction

With the development of artificial intelligence (AI) and increasing demand for big data in clinical medicine, it becomes particularly important to apply machine learning (ML) AI methods in clinical practice and research. The application of AI to specific critical diseases has far-reaching significance for guiding clinical treatment decisions and predicting clinical outcomes.

Neutropenia complicated with infection is an important complication in children with hematological and oncological disease. Low neutrophil count, chemotherapy- or radiotherapy-induced mucosal reaction and organ dysfunction, and central venous catheter placement are risk factors for concurrent infection in these patients. The incidence of septic shock in children with hematological and oncological disease accompanied by fever or neutropenia (FN) is 6%–7% (1, 2). Data from our center showed that the number of septic shock deaths in children with FN complicating hematological or oncological disease was 15 per year. Early recognition, early diagnosis, and early treatment of sepsis and septic shock are of vital importance, and early aggressive antibiotic and fluid resuscitation therapy is associated with better outcomes.

However, current guidelines related to FN in pediatric patients with hematological or oncological disease lack risk stratification and evaluation methods for sepsis and septic shock (2, 3). A clinical model using ML has been established and validated in the field of adult sepsis (4–7). However, there have been no reports on evaluating septic shock using ML-based septic shock early warning model (SSEW model) in FN children with hematological or oncological disease. In this study, we aimed to establish and preliminarily validate an AI septic shock prediction model (SSEW model, Feature engineering was shown in Additional file 1) for children with FN complicating hematological or oncological disease.

## Methods

### Participants

The subjects of our study were children with hematological or oncological disease hospitalized at the Department of Hematology and Oncology at Shanghai Children’s Medical Center from January 1, 2017 to August 1, 2019. Consecutive FN patients aged between 30 days and 18 years who were admitted to the hematologic oncology were included. Fever was defined as the temperature over 38°C, and neutropenia was defined as an absolute neutrophil count (ANC) lower than 1.0 × 10^9^/L. Exclusion criteria were as follows: 1. patients discharged automatically or died without diagnosis of septic shock; 2. patients who showed signs of septic shock already on admission; 3. patients whose data of case prediction features were incomplete.

### Design

Medical records of eligible patients were extracted from the electronic medical record (EMR) of the hospital. We used an ML algorithm to explore the risk factors for septic shock in pediatric inpatients with hematological and oncological disease, and the inputs for the model were selected based on the experts’ experience. Depending on the time of admission, a development set (from January 1, 2017 to January 1, 2019) and a validation set (from January 1, 2019 to August 1, 2019) were defined.

**Figure 1:**
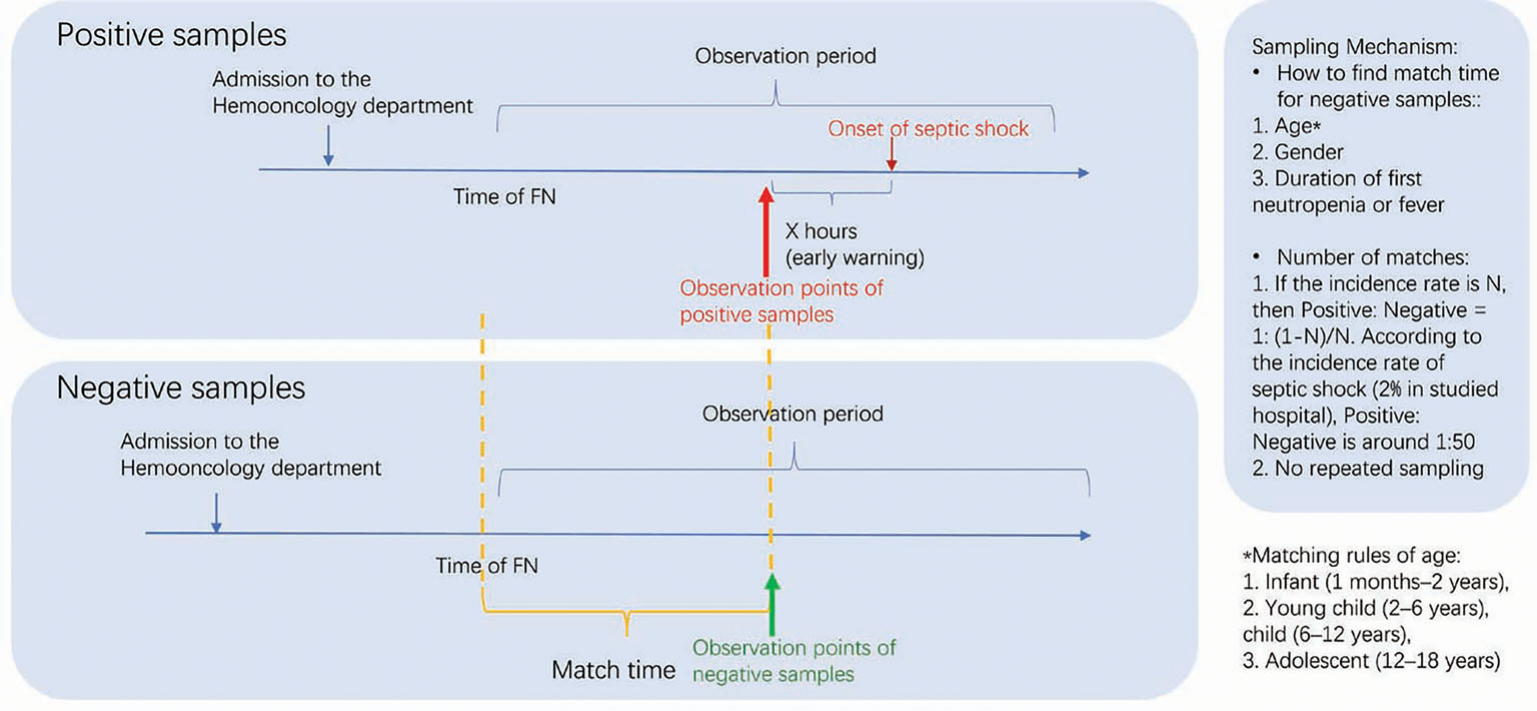
Diagram of episode sampling mode in the observation period.

Patients who met the inclusion criteria were included in the observation period, which started from FN and ended 72 hours after normalization of body temperature and recovery of ANC to 1 × 10^9^/L. The outcome labels of each sample were manually labeled retrospectively. The observation period of septic shock during hospitalization was defined as the septic shock group, and non-septic-shock group was the control group. The diagnosis of septic shock was based on the 2005 International guidelines for children with sepsis and septic shock (8). To ensure the quality of manual labeling in groups, this study was conducted by specialists of critical care and hematologic oncology. Each sample was independently marked by at least two physicians, and the samples with differences independently marked by two doctors were reviewed by a third doctor. Furthermore, observation points included 4, 8, 12, and 24 hours before shock in the septic shock group (OBS points), and corresponding observation points in the control group were obtained after matching with the septic shock group (**Figure 1**). This study was approved by the Ethics Committee of Shanghai Children’s Hospital Center affiliated to Shanghai Jiao Tong University Medical College (SCMCIRB-K2020046-1).

### Clinical Features

Various clinical features that could be extracted from the EMR system were included in this study as candidates for analysis, such as vital signs during admission (e.g., respiratory rate, body temperature, and pulse), laboratory test results (e.g., C-reactive protein level, neutrophil count, and platelet count), and treatment information. Full features can be found in **Supplementary Table 1**. Based on the OBS point of each hospitalization, the above features were expanded into the ML features pool according to certain rules. For example, white blood cell (WBC) count could be expanded to “the average WBC count at 8 h before OBS point” and “the maximum increase in WBC count at 16 h before OBS point”. About 1600 features were obtained by this method. Among them, features with >20% missing values were excluded from further analysis. The remaining boolean features and categorical features were filled as NA, and continuous features were filled with the mean value. Specific candidate features and the rules of expanding features into variables pools are shown in **Supplementary Material**.

### Machine Learning Modeling

In accordance with the general practice of building models in ML, we filtered the features before actual modeling. The details regarding this process are shown in **Supplementary Material**. During the observation period in accordance with inclusion criteria and exclusion criteria, the development set (from January 1, 2017 to January 1, 2019) and internal verification set (from January 1, 2019 to August 1, 2019) were defined depending on the time of hospitalization. In the development set, the filtered variable pool adopted Extreme gradient boosting (XGBoost) algorithm, and the model effect (discrimination) was measured by the area under receiver operating characteristic curve (AU-ROC) of features. This process was repeated in the internal validation set to validate the model. For comparison, traditional logistic regression modeling was performed synchronously, and pediatric sequential organ failure score (pSOFA) was calculated.

### Statistical Analysis

Continuous features were tested for normality by Anderson-Darling test. Depending on the normality of distribution, the data were represented by median (interquartile spacing, IQR) or mean (standard deviation, SD). Categorical features were expressed as frequencies. Chi-square test and Fisher’s exact probability test were used as applicable. P < 0.05 was considered to be statistically significant. The AU-ROC was used to evaluate the efficacy of different models or scores for early warning of septic shock.

## Results

### Participants

**Figure 2:**
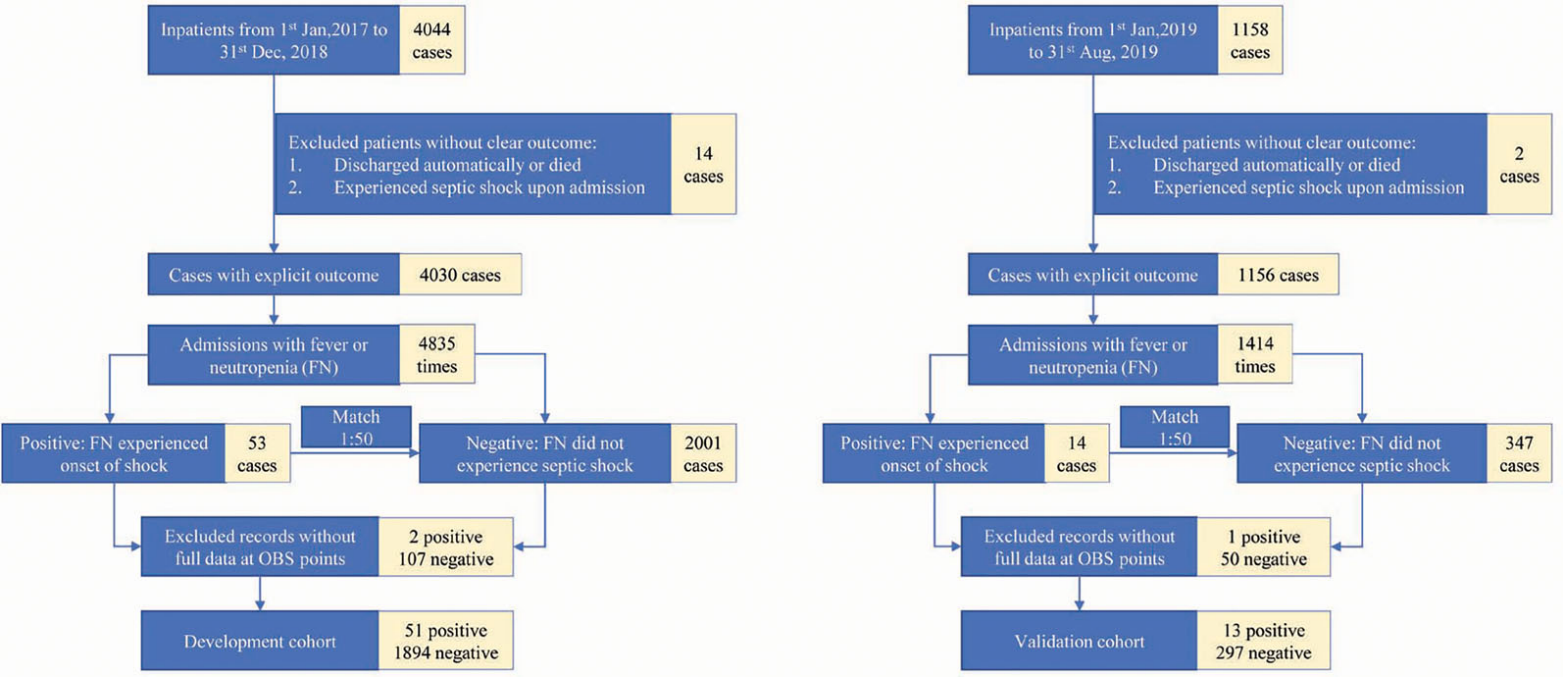
Flow chart of establishing development and validation cohorts.

A total of 1,238 children and 2,255 application periods were included. There were 64 observation periods in the septic shock group and 2,191 observation periods in the control group. Among them, the development set included 51 observation periods in the septic shock group and 1,894 observation periods in the control group, whereas the internal validation set included 13 observation periods in the septic shock group and 297 observation periods in the control group (**Figure 2**). The clinical features of the included children are shown in **Table 1**.

**Table 1:** Characteristics of 1,238 included patients.

Features	Characteristic
Gender (male), n (%)	738 (59.6)
Age (months), mean (IQR)	58.3 (25.8, 111.0)
Accepted bone marrow transplantation, n (%)	320 (25.9)
Accepted CAR-T, n (%)	24 (1.9)
Total admission time (days), mean (IQR)	22.0 (9.9,34.1)
Aplastic anemia, n (%)	133 (10.7)
Acute lymphoblastic leukemia, n (%)	444 (35.9)
Acute myeloid leukemia, n (%)	188 (15.2)
Neuroblastoma, n (%)	57 (4.6)
Non-Hodgkin lymphoma, n (%)	49 (4.0)
Mucopolysaccharidosis, n (%)	46 (3.7)
Solid malignant tumors, n (%)	121 (9.8)
Congenital immunodeficiency, n (%)	31 (2.5)
Hepatoblastoma, n (%)	28 (2.3)
Others, n (%)	139 (11.2)

### Choose of Optimal Model and OBS Points

**Figure 3:**
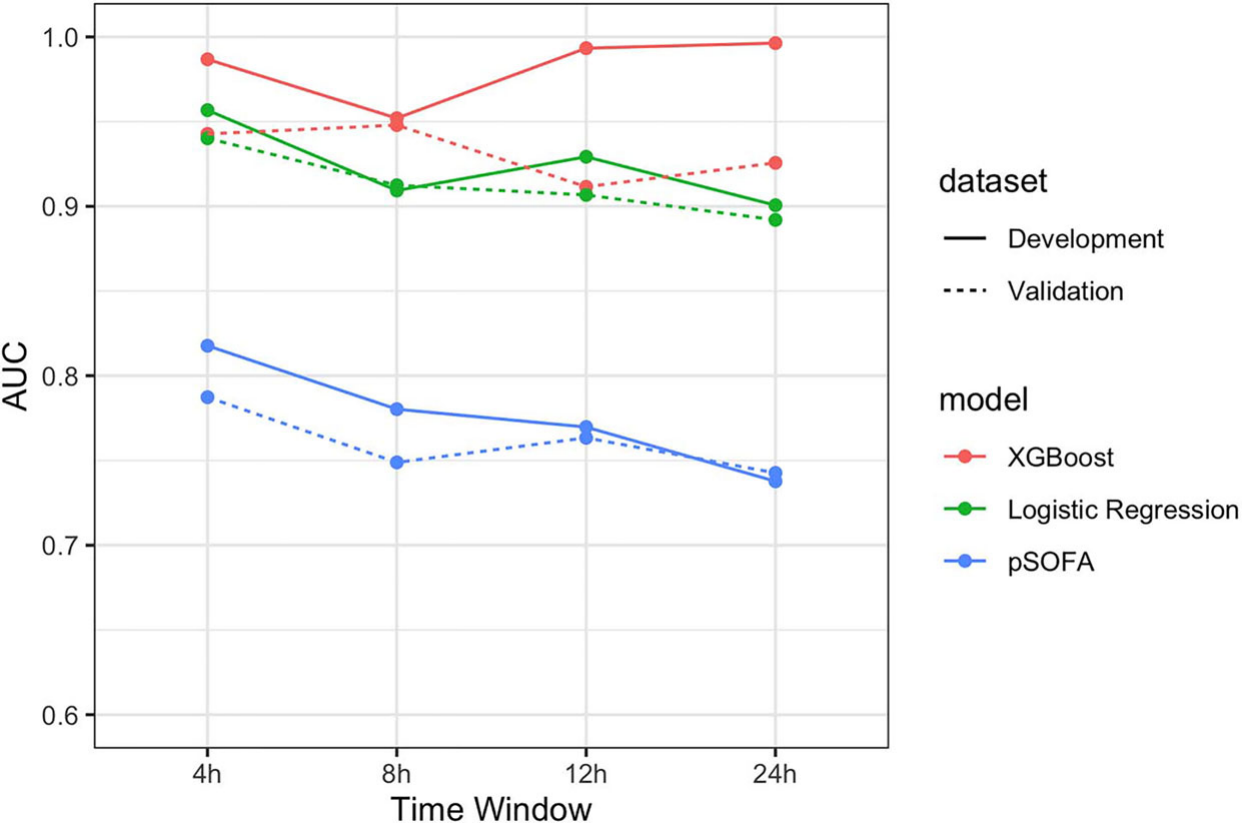
Comparison of AU-ROC between XGBoost, logistic regression, and pSOFA at observation points 4, 8, 12, and 24 h.

Comparison of ROC curve between XGBoost, logistic regression model, and pSOFA for septic shock at observation points 4, 8, 12, and 24 h is shown in **Figure 3**. In the validation set, the AU-ROC of XGBoost at observation points 4, 8, 12, and 24 h was superior to that of the pSOFA (**Figure 3**). Furthermore, the optimal OBS points was determined from two perspectives: the AU-ROC for septic shock as well as clinical usefulness. With a satisfying AUC, 24 hours before septic shock was chosen as the best time point to start early warning given it is also an ideal timing to take interventions to prevent sepsis shock.

### Model Performance at 24-h Observation Point

**Figure 4:**
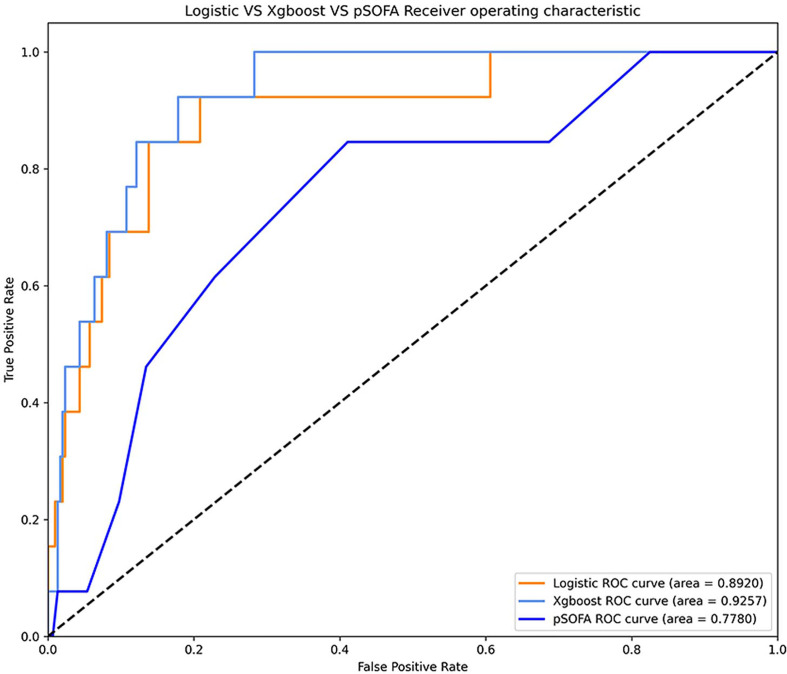
ROC curve of XGBoost model, logistic regression model, and pSOFA to predict septic shock.

Comparison of the ROC curves of XGBoost, logistic regression, and pSOFA at 24-h OBS point is shown in **Figure 4**. The AU-ROC of XGBoost model was superior to the pSOFA for predicting septic shock (0.93 vs. 0.76, Z = −2.73, P = 0.006).

### The SSEW Model

**Figure 5:**
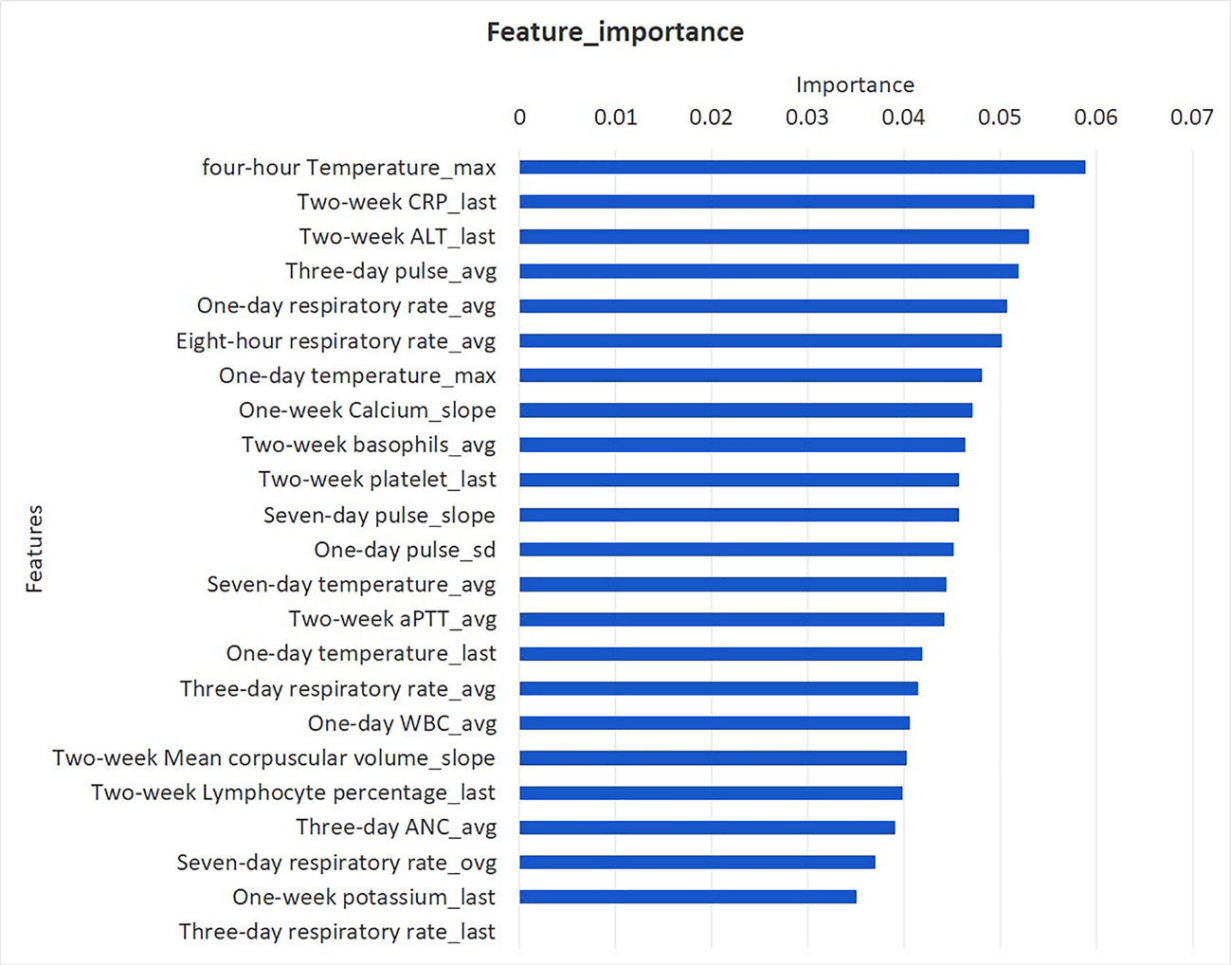
Feature importance derived from XGBoost model in 24 h OBS.

Comparison of root features between the septic shock group and control group at 24 h OBS point revealed that there was a total of 14 module root features. The differences between the groups are shown in **Table 2**. The 24 h observation point XGBoost incorporated 23 derived features, as shown in **Figure 5**. The final 23 features included in the model were classified as follows: vital signs (respiration, pulse, and body temperature), blood routine test values (WBC count, neutrophil count, platelet count, basophilic count, lymphocyte percentage, and mean hemoglobin volume), electrolytes (serum potassium and ionic calcium levels), liver function indicators (ALT level), coagulation function (APTT), and infection index (CRP).

**Table 2:** Characteristics between the groups at 24 h OBS point.

Characteristics	Septic shock group (n = 64)	Control group (n = 2191)	P-value
Ca^2+^ (mmol/L)	2.1 (1.9, 2.3)	2.3 (2.2, 2.4)	<0.001
K^+^ (mmol/L)	3.6 (3.1, 4.5)	4.3 (3.9, 4.7)	<0.001
Temperature (°C)	37.5 (36.9, 38.2)	37.0 (36.7, 37.4)	<0.001
RR (/min)	24.5 (22.8, 30.0)	23.0 (21.0, 25.0)	<0.001
WBC (×10^9^/L)	0.9 (0.1,3.0)	1.9 (0.9, 3.4)	<0.001
Pulse (/min)	122 (105.5, 134.0)	110 (100, 118)	<0.001
C-reactive protein (mg/L)	84.0 (15.5, 157.5)	11.0 (3.0, 36.0)	<0.001
Basophil count (×10^9^/L)	0 (0, 0.01)	0.01 (0, 0.02)	0.734
Mean corpuscular volume (fL)	87.2 (82.0, 91.1)	87.6 (82.9, 92.5)	0.387
APTT (s)	33.8 (30.4, 40.9)	33.2 (29.8, 37.2)	0.075
Lymphocyte percentage (%)	37.5 (6.8, 69.5)	38.5 (20.1, 64.3)	0.334
Platelet account (×10^9^/L)	22.0 (10.8, 77.0)	82.0 (34.0, 181.0)	<0.001
Alanine transaminase (U/L)	30.0 (16.8, 71.5)	28.0 (18.0, 47.0)	0.372
Neutrophil count (×10^9^/L)	0.4 (0.1, 2.8)	0.7 (0.2, 1.7)	0.434

## Discussion

With the development of AI and the increasing demand for big data in clinical medicine, it is particularly important to apply the AI tools in clinical practice and research. It has been reported that an AI ML-based early warning model can improve the early detection and timely treatment of patients with sepsis, and it can predict the possibility of septic shock (6, 9–11). Wang et al. (12) used simple EMR features, such as WBC count, heart rate, and Acute Physiology and Chronic Health Score 2 (APACHE-2), to model severity of sepsis in intensive care unit patients, and achieved an AU-ROC of 0.94 for predicting severe sepsis. However, their study did not include patients with any hematological malignancies with neutropenia or immunosuppression.

Infection is one of the main causes of death in patients with FN. The incidence rate of sepsis in children aged 1-9 years with hematological malignancies was 12.8% (13), of which 69% cases were complicated with severe sepsis and septic shock (14). Septic shock associated with hematologic malignancies with fever or neutropenia is more likely to occur than that without underlying disease. Therefore, the population selected in this study included pediatric patients with hematological and oncological disease who developed fever or neutropenia during the treatment of underling disease.

In this study, we preliminarily established an SSEW model based on EMR machine learning and XGBoost algorithm for early warning of septic shock in FN pediatric patients with hematological or oncological disease. The modeling process of this study involved many features that are repeatedly measured at multiple time points. In order to better capture the information of the change of these features over time, we aggregated the features within a certain time window to derive the characteristics such as maximum, minimum, average, last value, earliest value, average development speed, standard deviation, and maximum increase (9, 15). We obtained 14 root features at the 24-h observation point, and combined the original features and derived features together with ML to finally include 23 features. In the process of variable screening, we adopted simulated annealing algorithm, which is suitable for solving mathematical problems abstracted by variable screening. According to the principle of simulated annealing algorithm, this algorithm is one of the global search algorithms and has the characteristics of good generality. Especially compared with stepwise regression, it can greatly save calculation time and find the optimal solution as soon as possible. The final 23 features included in the model were classified as follows: vital signs (respiration, pulse, and body temperature), blood routine test values (WBC count, neutrophil count, platelet count, basophilic count, lymphocyte percentage, and mean hemoglobin volume), electrolytes (serum potassium and ionic calcium levels), liver function indicators (ALT level), coagulation function (APTT), and infection index (CRP). According to the classification, our model dynamically evaluated vital signs, myelosuppression, infection indicators, liver function, and the state of the internal environment in febrile and granulocytopenia patients with hematologic tumors.

In this study, pSOFA score was used to compare its predictive efficacy for septic shock warning with the SSEW model. The pSOFA score focuses on the functional status of organs in children with sepsis and septic shock. The 2016 International Save Sepsis Campaign proposed the Third International Consensus Definitions for Sepsis and Septic Shock (Sepsis-3) as a life-threatening organ dysfunction caused by a dysregulated host response to infection (16). Sepsis-3 used the Sequential Organ Failure Assessment (SOFA) to reach the diagnosis of sepsis. For febrile patients with granulocytosis, SOFA or quick SOFA (qSOFA) are still recommended for the diagnosis of sepsis. The 2020 Surviving Sepsis Campaign International Guidelines for the Management of Septic Shock and Sepsis-Associated Organ Dysfunction in Children has already mentioned the definition of organ dysfunction associated with childhood sepsis (2). However, SOFA is based on adult data, and normal values of organ functions vary between children of different ages (17–19). Therefore, the new guidelines still do not sublimate the definition of pediatric sepsis to Sepsis-3. In 2017, Matics et al. (20) modified the SOFA score on the basis of the age stratification method of PELOD-2 scoring for central vascular system and renal function indexes, and according to the age stratification index of SpO_2_/FiO_2_ for assessing lung injury in children (21). Consequently, they proposed the Pediatric SOFA (pSOFA) score, which encompasses six indicators stratified by age, including oxygenation index (SpO_2_/FiO_2_), platelet count, bilirubin, mean arterial pressure, Glasgow score, and serum creatinine score.

At the 24-h observation point, the root variable shared by the SSEW model, logistics model, and pSOFA was platelet count. The target population of pSOFA score includes all possible sepsis in critically ill children, and the patients included in this study belonged to a specific group with hematologic tumors associated with fever or cytopenia. Moon et al. (22) reported that laboratory indicators such as platelet count < 50 × 10^9^/L, serum CRP level > 10 mg/dL, and chest X-ray lung infiltration are independent factors for predicting complications in neutropenic fever patients in emergency department (ED). Lynn et al. (23) have also shown the association of delayed use of antibiotics for pneumonia and platelet count ≤ 50 × 10^9^/L with severe complications in chemotherapy-induced febrile neutropenia patients in ED setting. Considering that the SSEW model, logistics model, and pSOFA included platelet count as one of the features, we concluded that platelet count was not only associated with the organ function in septic shock, but also reflected the situation of bone marrow suppression after chemotherapy in children with hematological tumor, which was closely related to the occurrence of septic shock in these patients.

At the 24-h observation point, we demonstrated that neutrophil count was another root variable commonly included by the SSEW model and logistic regression model. The effect of neutropenia on the occurrence and prognosis of sepsis and septic shock remains controversial. Children with hematologic tumors need to receive combined chemotherapy. Neutropenia can last for several weeks, and severe infection is related to the degree and duration of neutropenia (24–26). In a large meta-analysis of more than 1700 critically ill patients with neutropenia, neutropenia was independently associated with adverse outcomes (27). However, patients with neutropenic sepsis may have atypical clinical manifestations. In patients with normal bone marrow function, when infection occurs, the body experiences an inflammatory and immune response involving WBS and lymphocytes produced by the bone marrow (28). In contrast, patients with a deficiency in WBC and lymphocytes may show suppressed inflammation and immune response, so that the usual symptoms of infection may disappear. In a retrospective study, Soares et al. pointed out that oncologists and intensive care physicians should make treatment plans and implement protocols based on sepsis guidelines on a daily basis, so that lower hospital mortality in critically ill patients with tumor is ensured. Through the establishment of the SSEW model and logistics models, we concluded that neutrophil count was particularly important for the early warning of septic shock in children with hematologic tumor FN.

The results of this study showed that the SSEW model was superior to pSOFA for early warning of septic shock in FN children with hematology and oncology at 4, 8, 12, and 24-h observation points. The first possible reason was that adult sepsis research has shown that qSOFA score is not accurate as a screening tool for chemotherapy-induced FN with sepsis, mortality, and ICU admission (29, 30). There are several important factors that should be considered in using qSOFA for evaluating the possibility of septicemia in FN adult cancer patients. As these patients have fever, they are suspected of infection after contact; however, due to immunosuppression, their ability to produce inflammatory response is reduced, which can limit the signs and symptoms of infection (31). Therefore, fever may be the only abnormal manifestation, leading to false negative results. We should consider the possibility of different forms of organ dysfunction that have not yet been included in qSOFA and thereby reduce its sensitivity. In addition, patients with qSOFA ≥ 2 and no sepsis should also be considered, because decreased oral dose, dehydration, and frailty in cancer patients may lead to abnormal vital signs, including hypotension. Regardless of the severity of the infection, the extent of the disease, such as brain metastasis or pial metastasis, and opioid drugs may cause changes in the condition, which are included in the qSOFA criteria. The second possible reason was that the SSEW model included more root features than pSOFA score, and these features such as CRP and temperature were particularly important for the development of septic shock. CRP is one of the biomarkers of sepsis and septic shock infection, and Sano et al. marked it as a risk factor for sepsis-related death in children with hematologic and malignant diseases. Therefore, we included the features related to leukocytes, neutrophils, and CRP in the model, and the results of this study were consistent with those reported in the literature. After artificial learning and obtaining the variation trend of these features, we were able to more accurately predict the occurrence of septic shock. The traditional definition of fever in patients with neutropenic sepsis is a temperature greater than 38.3°C or lasting ≥ 1 h (32). However, in patients with neutropenic sepsis, body temperature may be within the normal range or even in the range of hypothermia due to immune deficiency or the use of cortisol medications (33). Therefore, a body temperature below 36°C may also be an indicator of severe sepsis (34).

The third possible reason why the SSEW model was superior to pSOFA in predicting septic shock in FN children with hematology and oncology at 4, 8, 12, and 24 h observation points is that it aggregates the features within a certain time window, thus deriving features such as maximum, minimum, average, latest, earliest, average development speed, standard deviation, and maximum increase. For example, 14 root features are derived into 23 derived features in the SSEW model. For body temperature, three derivative features including the mean body temperature within 7 days, the maximum body temperature within 1 day, and the most recent body temperature within 1 day were included in the SSEW model. Compared with the traditional single temperature measurement, it is obviously more accurate to find the predictive value of normal temperature or hypothermia for the occurrence of septic shock in such children. The results showed that the SSEW model compared with the traditional early warning method targets different basic diseases in children and can be more accurate and precise in screening the predictive features for specific diseases. In addition, the SSEW model reflects specific features and dynamic changes during the occurrence of septic shock through derived features, and finally provides the early warning ability for children with septic shock.

This study had some advantages and drawbacks. The advantage was that the SSEW model algorithm in this study used XGBoost, which has been used already in predicting the failure of treatment for parapneumonic empyema and the volumetric responsiveness of acute kidney injury (AKI) (35, 36); indeed, the predictive accuracy of the XGBoost model was significantly better than the one of a generalized linear model. Since the XGBoost model is an ensemble of weak prediction trees, it is able to capture complex relationships in the data without the need to be explicitly specify high-order interactions and non-linear functions. There were several limitations. First, this study retrospectively analyzed the EMR data, which were not originally designed for analysis. However, we cannot deny that these data demonstrated practicality in a clinical setting in which children with hematologic tumors are hospitalized, and showed good performance even in the absence of data. Second, as this was a retrospective study, the method of manual labeling by medical experts was adopted in the diagnosis of septic shock. Finally, EMR data from hematologic oncology wards rather than ICUs may limit the future use of SSEW models in ICUs and external validation. However, these flawed data points are suitable for use in our model, which contains predictive information when combined with other measurements through machine learning.

## Conclusion

Early warning of septic shock in FN children with hematological or oncological disease is an important clinical need. This study showed that the AI-based SSEW model for septic shock in FN children with hematological or oncological disease was efficient in early warning at 4, 8, 12, and 24 h, and it was better than the pSOFA score. Hence, it may help clinicians to identify the risk of developing septic shock in children even 24 h in advance, thereby helping to achieve early clinical monitoring and treatment of septic shock. In the future, it would be necessary to conduct prospective studies based on clinical application scenarios to determine the clinical utility of this predictive SSEW model.

## Data Availability Statement

The raw data supporting the conclusions of this article will be made available by the authors, without undue reservation.

## Ethics Statement

This study was approved by the Ethics Committee of Shanghai Children’s Hospital Center affiliated to Shanghai Jiao Tong University Medical College (SCMCIRB-K2020046-1). The informed consent requirement was waived because the study only involved the use of past clinical data.

## Author Contributions

YW, LZ, LF, and LX took responsibility of literature search, study design, writing and critical revision. HW, SF, WZ, HL, BD, SL, and YC mainly participated in data collection, data analysis and data interpretation. All authors contributed to the article and approved the submitted version.

## Funding

The study was supported by Pudong New Area Science and Technology Development Fund (Grant number: PKX2019-R06), the Science and Technology Innovation-Biomedical Supporting Program of Shanghai Science and Technology Committee [Grant number: 19441904400] and Program for Artificial Intelligence Innovation and Development of Shanghai Municipal Commission of Economy and Informatization.

## Conflict of Interest

Authors SF and YC were employed by the company Shanghai Synyi Medical Technology CO. Ltd.

The remaining authors declare that the research was conducted in the absence of any commercial or financial relationships that could be construed as a potential conflict of interest.

## Supplementary Material

Supplementary Figure 1 Feature engineering (variable aggregation and selection) in SSEW model.
