# Supplementary material for: Machine Learning for Early Warning of Septic Shock in Children With Hematological Malignancies Accompanied by Fever or Neutropenia: A Single Center Retrospective Study
Source: Front Oncol. 2021 Jun 15;11:678743. doi: 10.3389/fonc.2021.678743 (PMC8240637; doi:10.3389/fonc.2021.678743)
Supplement: Supplementary Figure 1 — Feature engineering (variable aggregation and selection) in SSEW model. [file DataSheet_1.docx]

Feature engineering in SSEW model

Feature engineering is the process of creating effective features from data sources to establish SSEW machine learning models (37). A delicate feature engineering system could accelerate modeling and improve predictive performance significantly. We included two components in the process: feature aggregation and feature selection, based on root features we included (Supplementary Table 1).

In the feature aggregation component, three factors had to be specified to derive features: root variables, time windows, and aggregation methods. We retrieved all values for a root variable within a time window before OBS time from electronic medical records and then derived features of statistics. Time window setup included 4, 8, and 12 hours, and 1, 3, and 7 days, while aggregation methods included average (avg), slope, last, maximum (max), minimum (min), and standard deviation (sd). For example, as shown in Supplementary figure 1, respiratory rate can be aggregated as an average respiratory rate 4 hours before OBS points or the maximum respiratory rate 8 hours before OBS points. In this example, we achieved 36 features related to respiratory rate. As a result of feature aggregation, 1600 features were obtained in the candidate variable pool.

After generating variables, the feature selection component was executed, including information value (IV) filter, correlation filter, and simulated annealing (SA) filter. The first step was to evaluate the discrimination of all features by IV (38). A feature was kept if its IV value was larger than 0.1. The second step was to compute the absolute values of Pearson correlation coefficients to measure redundancy of the remaining features from the IV filter (39). If feature redundancy between a pair was larger than 0.6, then the one with lower IV was eliminated in the correlation filter. The last step was based on a filter-wrapper hybrid strategy (40). Briefly speaking, a random forest classifier was trained to obtain feature importance for all features; then, a decision tree classifier was trained for each feature to obtain single feature F1 score. These two indicators measured the union and single distinguishing ability of features. Subsequently, a simulated annealing algorithm was used to seek the optimal threshold values for the two indicators as well as binary receiving indicators of features by means of an additional random forest classifier. All machine learning models were trained in a cross-validation manner on the developing dataset. For example, among all the derived 36 features of respiratory rate (as shown in Fig), 28, 10, and 5 features were kept sequentially in the steps of IV, correlation, and SA filters, respectively.

Using this feature engineering strategy, 23 variables were finally selected for further statistical analysis and machine learning modeling.

Reference:

37. Domingos P. A few useful things to know about machine learning. Commun ACM. 2012;55(10):78–87.

38. Scorecard Development Process, Stage 4: Scorecard Development. Credit Risk Scorecards2012. p. 73-130.

39. Schober P, Boer C, Schwarte LA. Correlation Coefficients: Appropriate Use and Interpretation. Anesthesia & Analgesia. 2018;126(5):1763-8.

40. Li-Xin Z, Jia-Xin W, Yan-Nan Z, Ze-Hong Y, editors. A novel hybrid feature selection algorithm: using ReliefF estimation for GA-Wrapper search. Proceedings of the 2003 International Conference on Machine Learning and Cybernetics (IEEE Cat No03EX693); 2003 5-5 Nov. 2003.

Supplementary Table 1. Root Features And Time Windows For Feature Aggregation

| **Root Features** | **Time Window (from OSB）** | **Aggregation** |
| --- | --- | --- |
| Age | NA | last |
| Gender | NA | last |
| Height | NA | last |
| Weight | NA | last |
| Systolic Blood Pressure | -4h,-8h,-12h,-1d,-3d,-7d | avg,slope,last,max,min,sd |
| Diastolic Blood Pressure | -4h,-8h,-12h,-1d,-3d,-7d | avg,slope,last,max,min,sd |
| Heart Rate | -4h,-8h,-12h,-1d,-3d,-7d | avg,slope,last,max,min,sd |
| Pulse | -4h,-8h,-12h,-1d,-3d,-7d | avg,slope,last,max,min,sd |
| Respiratory Rate | -4h,-8h,-12h,-1d,-3d,-7d | avg,slope,last,max,min,sd |
| Temperature | -4h,-8h,-12h,-1d,-3d,-7d | avg,slope,last,max,min,sd |
| SpO2 | -4h,-8h,-12h,-1d,-3d,-7d | avg,slope,last,max,min,sd |
| Procalcitonin | -1d,-2d,-3d,-4d,-1w,-2w | avg,slope,last |
| Aspartate Transaminase | -1d,-2d,-3d,-4d,-1w,-2w | avg,slope,last |
| Alkaline Phosphatase | -1d,-2d,-3d,-4d,-1w,-2w | avg,slope,last |
| Glutamyltransferase | -1d,-2d,-3d,-4d,-1w,-2w | avg,slope,last |
| Total Bilirubin | -1d,-2d,-3d,-4d,-1w,-2w | avg,slope,last |
| Direct Bilirubin | -1d,-2d,-3d,-4d,-1w,-2w | avg,slope,last |
| Total Protein | -1d,-2d,-3d,-4d,-1w,-2w | avg,slope,last |
| Albumin | -1d,-2d,-3d,-4d,-1w,-2w | avg,slope,last |
| Globulin | -1d,-2d,-3d,-4d,-1w,-2w | avg,slope,last |
| Albumin/Globulin | -1d,-2d,-3d,-4d,-1w,-2w | avg,slope,last |
| Serum Urea Nitrogen | -1d,-2d,-3d,-4d,-1w,-2w | avg,slope,last |
| Creatinine | -1d,-2d,-3d,-4d,-1w,-2w | avg,slope,last |
| Uric Acid | -1d,-2d,-3d,-4d,-1w,-2w | avg,slope,last |
| Alanine Aminotransferase | -1d,-2d,-3d,-4d,-1w,-2w | avg,slope,last |
| Syphilis Screening（+） | NA | any |
| Prothrombin Time | -1d,-2d,-3d,-4d,-1w,-2w | avg,slope,last |
| Activated Partial Thromboplastin Time | -1d,-2d,-3d,-4d,-1w,-2w | avg,slope,last |
| Thrombin Time | -1d,-2d,-3d,-4d,-1w,-2w | avg,slope,last |
| Fibrinogen | -1d,-2d,-3d,-4d,-1w,-2w | avg,slope,last |
| D-Dimer | -1d,-2d,-3d,-4d,-1w,-2w | avg,slope,last |
| International Normalized Ratio | -1d,-2d,-3d,-4d,-1w,-2w | avg,slope,last |
| Fibrin Degradation Products | -1d,-2d,-3d,-4d,-1w,-2w | avg,slope,last |
| Blood Lactic Acid | -1d,-2d,-3d,-4d,-1w,-2w | avg,slope,last |
| C-Reactive Protein | -1d,-2d,-3d,-4d,-1w,-2w | avg,slope,last |
| White Blood Cell Count | -1d,-2d,-3d,-4d,-1w,-2w | avg,slope,last |
| Lymphocyte % | -1d,-2d,-3d,-4d,-1w,-2w | avg,slope,last |
| Monocytes % | -1d,-2d,-3d,-4d,-1w,-2w | avg,slope,last |
| Neutrophil % | -1d,-2d,-3d,-4d,-1w,-2w | avg,slope,last |
| Basophil % | -1d,-2d,-3d,-4d,-1w,-2w | avg,slope,last |
| Eosinophil % | -1d,-2d,-3d,-4d,-1w,-2w | avg,slope,last |
| Lymphocyte Count | -1d,-2d,-3d,-4d,-1w,-2w | avg,slope,last |
| Monocyte Count | -1d,-2d,-3d,-4d,-1w,-2w | avg,slope,last |
| Neutrophils Count | -1d,-2d,-3d,-4d,-1w,-2w | avg,slope,last |
| Basophil Count | -1d,-2d,-3d,-4d,-1w,-2w | avg,slope,last |
| Eosinophil Count | -1d,-2d,-3d,-4d,-1w,-2w | avg,slope,last |
| Red Blood Cells Count | -1d,-2d,-3d,-4d,-1w,-2w | avg,slope,last |
| Hemoglobin Determination | -1d,-2d,-3d,-4d,-1w,-2w | avg,slope,last |
| Mean Erythrocyte Volume | -1d,-2d,-3d,-4d,-1w,-2w | avg,slope,last |
| Mean Corpuscular Hemoglobin | -1d,-2d,-3d,-4d,-1w,-2w | avg,slope,last |
| Mean Corpuscular Hemoglobin Concentration | -1d,-2d,-3d,-4d,-1w,-2w | avg,slope,last |
| Red Cell Distribution Width | -1d,-2d,-3d,-4d,-1w,-2w | avg,slope,last |
| Platelet Count | -1d,-2d,-3d,-4d,-1w,-2w | avg,slope,last |
| Mean Platelet Volume | -1d,-2d,-3d,-4d,-1w,-2w | avg,slope,last |
| Platelet Volume Distribution Width | -1d,-2d,-3d,-4d,-1w,-2w | avg,slope,last |
| Interleukin-2 | -1d,-2d,-3d,-4d,-1w,-2w | avg,slope,last |
| Interleukin-6 | -1d,-2d,-3d,-4d,-1w,-2w | avg,slope,last |
| Interleukin-8 | -1d,-2d,-3d,-4d,-1w,-2w | avg,slope,last |
| Interleukin-10 | -1d,-2d,-3d,-4d,-1w,-2w | avg,slope,last |
| Tumor Necrosis Factor-Α | -1d,-2d,-3d,-4d,-1w,-2w | avg,slope,last |
| Pondus Hydrogenii ( | -1d,-2d,-3d,-4d,-1w,-2w | avg,slope,last |
| Partial Pressure of Carbon Dioxide | -1d,-2d,-3d,-4d,-1w,-2w | avg,slope,last |
| Partial Pressure of Oxygen | -1d,-2d,-3d,-4d,-1w,-2w | avg,slope,last |
| Oxygen Saturation | -1d,-2d,-3d,-4d,-1w,-2w | avg,slope,last |
| K+ | -1d,-2d,-3d,-4d,-1w,-2w | avg,slope,last |
| Na+ | -1d,-2d,-3d,-4d,-1w,-2w | avg,slope,last |
| Ca+ | -1d,-2d,-3d,-4d,-1w,-2w | avg,slope,last |
| Cl- | -1d,-2d,-3d,-4d,-1w,-2w | avg,slope,last |
| Glu | -1d,-2d,-3d,-4d,-1w,-2w | avg,slope,last |
| Lactic Acid | -1d,-2d,-3d,-4d,-1w,-2w | avg,slope,last |
| HCO3- | -1d,-2d,-3d,-4d,-1w,-2w | avg,slope,last |
| Standard Base Excess | -1d,-2d,-3d,-4d,-1w,-2w | avg,slope,last |
| Anion Gap | -1d,-2d,-3d,-4d,-1w,-2w | avg,slope,last |
| Hemoglobin | -1d,-2d,-3d,-4d,-1w,-2w | avg,slope,last |
| Red Blood Cell Volume | -1d,-2d,-3d,-4d,-1w,-2w | avg,slope,last |
| Oxyhemoglobin | -1d,-2d,-3d,-4d,-1w,-2w | avg,slope,last |
| Deoxygenated Hemoglobin | -1d,-2d,-3d,-4d,-1w,-2w | avg,slope,last |
| Temperature Corrected PCO2 | -1d,-2d,-3d,-4d,-1w,-2w | avg,slope,last |
| Temperature Correction Oxygen Partial Pressure | -1d,-2d,-3d,-4d,-1w,-2w | avg,slope,last |
| Oxygen Content | -1d,-2d,-3d,-4d,-1w,-2w | avg,slope,last |
| Partial Pressure of Oxygen At Half Saturation | -1d,-2d,-3d,-4d,-1w,-2w | avg,slope,last |
| Plasma Bicarbonate | -1d,-2d,-3d,-4d,-1w,-2w | avg,slope,last |
| Urine Colour | -1d,-2d,-3d,-4d,-1w,-2w | cat |
| Transparency | -1d,-2d,-3d,-4d,-1w,-2w | cat |
| Urine PH | -1d,-2d,-3d,-4d,-1w,-2w | avg,slope,last |
| Urine White Blood Cell | -1d,-2d,-3d,-4d,-1w,-2w | any |
| Urinary Nitrite | -1d,-2d,-3d,-4d,-1w,-2w | cat |
| Proteinuria | -1d,-2d,-3d,-4d,-1w,-2w | cat |
| Glycosuria | -1d,-2d,-3d,-4d,-1w,-2w | cat |
| Ketone | -1d,-2d,-3d,-4d,-1w,-2w | cat |
| Urobilinogen | -1d,-2d,-3d,-4d,-1w,-2w | cat |
| Urine Bilirubin | -1d,-2d,-3d,-4d,-1w,-2w | cat |
| Urine Erythrocyte/Red Blood Cell | -1d,-2d,-3d,-4d,-1w,-2w | any |
| Particle Shape | -1d,-2d,-3d,-4d,-1w,-2w | any |
| Crystallization | -1d,-2d,-3d,-4d,-1w,-2w | any |
| Bone Marrow Transplantation | NA | any |
| Myelodysplastic Syndromes | NA | any |
| Leukemia | NA | any |
| Lymphoma | NA | any |
| Pneumonia | NA | any |
| Stem Cell Transplantation | NA | last |
| PICC Duration | NA | last |
| Penicillin and Compound Preparation Antibiotics | NA | any |
| Cephalosporin Antibiotics | NA | any |
| Macrolide Antibiotics | NA | any |
| Aminoglycoside Antibiotics | NA | any |
| Carbapenem Antibiotics | NA | any |
| Zolidinone Antibiotics | NA | any |
| Peptide Antibiotics | NA | any |
| Sulfonamides Antibiotics | NA | any |
| 4-Quinolones | NA | any |
| Antifungal Agent | NA | any |
| Immunosuppressive Agent | NA | any |
| Glucocorticoid | NA | any |
| Angiotensin | NA | any |
| Vasodilators | NA | any |
| Cardiotonic | NA | any |
| Stimulating Factor | NA | any |
| HCV-IgG | NA | any |
| AIDS (+) | NA | any |
| HBSAg | NA | any |
| HBSAb | NA | any |
| HBEAg | NA | any |
| HBEAb | NA | any |
| HBCAb | NA | any |
| PICC | NA | any |
| Chemotherapy Pump Usage | NA | any |
| Aplastic Anemia | NA | any |
| Drug Cooling | -1d | count |
| Physical Cooling | -1d | count |
| Antibiotic Combination | NA | count |

Avg: Average; Max: Maximum; Min: Minimum; Sd: Standard Deviation


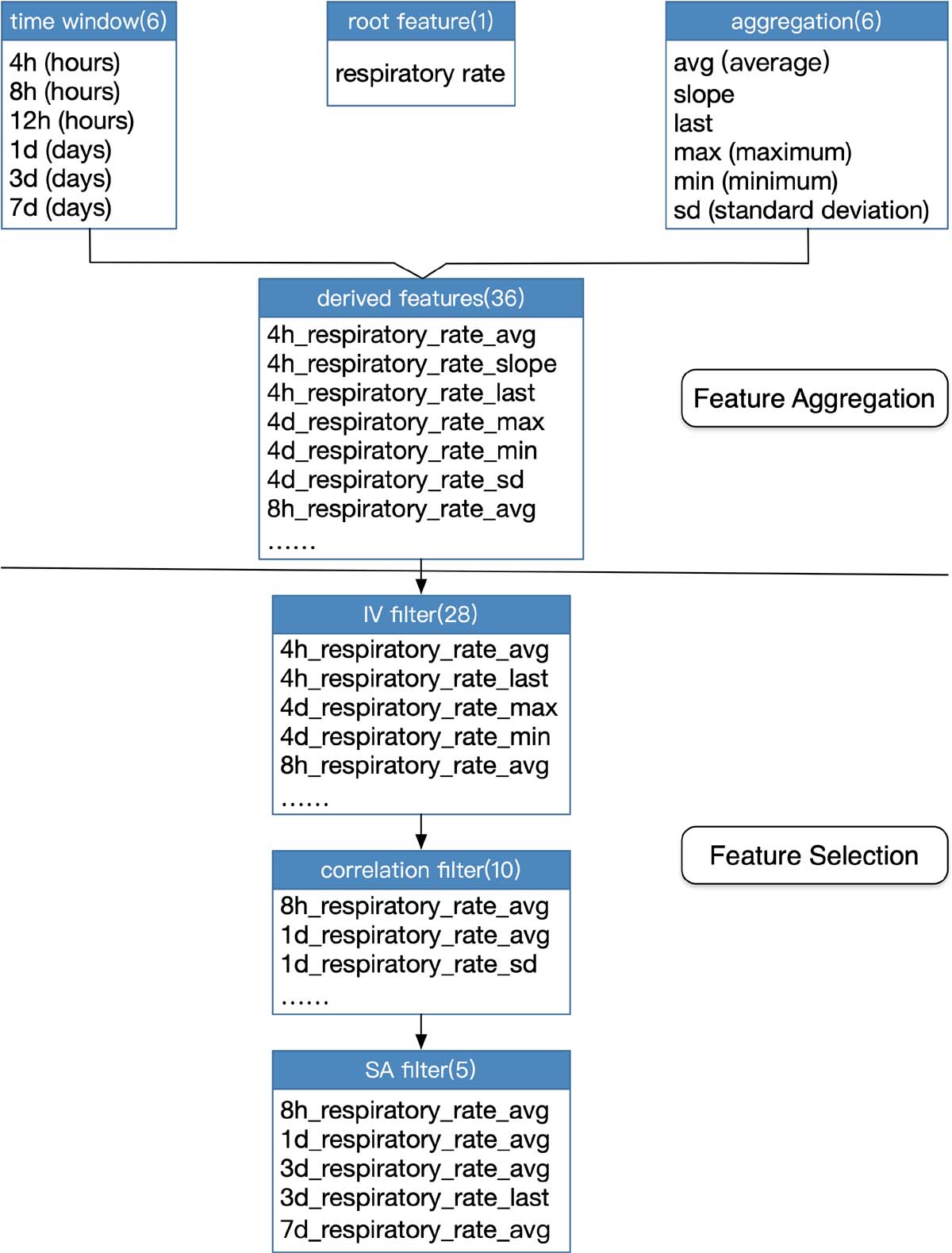


Supplement Figure 1. Feature engineering (variable aggregation and selection) in SSEW model

IV: information value; SA: simulated annealing
